# A sound effect: Exploration of the distinctiveness advantage in voice recognition

**DOI:** 10.1002/acp.3424

**Published:** 2018-07-04

**Authors:** Sarah V. Stevenage, Greg J. Neil, Beth Parsons, Abi Humphreys

**Affiliations:** ^1^ University of Southampton Department of Psychology Southampton UK; ^2^ Southampton Solent University School of Sport, Health and Social Sciences Southampton UK; ^3^ University of Winchester Department of Psychology Winchester UK

**Keywords:** distinctiveness advantage, temporal reversal, voice recognition

## Abstract

Two experiments are presented, which explore the presence of a distinctiveness advantage when recognising unfamiliar voices. In Experiment 1, distinctive voices were recognised significantly better, and with greater confidence, in a sequential same/different matching task compared with typical voices. These effects were replicated and extended in Experiment 2, as distinctive voices were recognised better even under challenging listening conditions imposed by nonsense sentences and temporal reversal. Taken together, the results aligned well with similar results when processing faces, and provided a useful point of comparison between voice and face processing.

## 1 INTRODUCTION

The capacity to recognise someone from their face is relatively well researched in terms of theoretical, behavioural, and neuropsychological findings (see Schweinberger & Burton's special issue, 2011). Against this backdrop, attention has relatively recently turned to the area of voice recognition. In this regard, researchers have been keen to identify similarities in performance between voice and face processing through applying the theories and methodologies from one area to the study of the other area. The present paper draws on this approach with specific focus on the parallel effects of distinctiveness in the face recognition and voice recognition literatures.

### 1.1 Recognising faces and recognising voices

The voice and the face are perhaps the two most readily available cues to identity (Ellis, Jones, & Mosdell, 1997). Both provide rich sources of information, communicating both affective state, and linguistic speech information as well as identity. Indeed, the voice has become known as an “auditory face” (Belin, Bestelmeyer, Latinus, & Watson, 2011; Belin, Fecteau, & Bédard, 2004) in recognition of the range of valuable information that it can indicate. With both faces and voices providing complementary cues about an individual, it is tempting to hold similar expectations when considering their processing. Despite this, early consideration suggested that voice recognition was substantially weaker than face recognition (see Stevenage & Neil, 2014 for a review). Notably, when recognising famous celebrities, voice recognition was significantly worse than face recognition, and voices produced significantly more “familiar only” experiences compared with faces (Ellis et al., 1997; Hanley, Smith, & Hadfield, 1998). In fact, a series of well‐designed studies suggested that voice and face recognition could only be equated when face recognition was compromised through substantial levels of blurring (Damjanovic & Hanley, 2007; Hanley & Damjanovic, 2009). Voices also served as weaker cues relative even to blurred faces when trying to retrieve both semantic details about celebrities, such as their occupation (Hanley et al., 1998; Hanley & Damjanovic, 2009), and episodic details about a time when they were previously encountered (Damjanovic & Hanley, 2007).

One possible explanation for the relative weakness of voices compared with faces, both when recognising celebrities and when retrieving information about them, is that participants may have experienced greater exposure to faces than voices given the popularity of media images. To test this account, Brédart and colleagues examined performance with personally familiar stimuli (which were likely to be heard as often as seen) and newly learned stimuli (for which face and voice exposure could be carefully controlled). As above, the results suggested that voices led to poorer retrieval of semantic details than faces, both when stimuli were personally familiar (Barsics & Brédart, 2011; Brédart, Barsics, & Hanley, 2009) and when newly learned (Barsics & Brédart, 2012a). Voices also led to poorer retrieval of episodic information compared with faces when stimuli were personally familiar (Barsics & Brédart, 2011). The fact that performance was still poorer in these studies when cued with voices than faces suggested that differential exposure was unlikely to account for the previous findings (Brédart & Barsics, 2012). Instead, the results suggested that, although both voices and faces could be used as cues to identity, the voice was less effective compared with the face.

This conclusion has been supported by results from several convergent methodologies. Using a priming methodology, for example, cross‐modal repetition priming has been demonstrated, whereby the face of a celebrity target facilitated the later recognition of their voice, and vice versa (Ellis et al., 1997; Schweinberger, Herholz, & Stief, 1997). However, the results of Stevenage, Hugill, and Lewis (2012) suggested that the voice was a far weaker prime for later face recognition than the face was for later voice recognition. In an adaptation to this task, a conflicting voices paradigm was developed in which celebrity recognition was examined from the face and voice under conditions in which the face and voice either matched (both belonged to the same celebrity) or mismatched (both belonged to different celebrities). Performance indicated that face recognition remained strong and robust regardless of the identity of the accompanying voice. However, voice recognition was substantially impaired when the accompanying face belonged to a different celebrity (Stevenage, Neil, & Hamlin, 2014).

Finally, the results of interference studies are relevant. In this paradigm, distractor faces were presented in between the study and test phases of a face‐matching task, and distractor voices were presented in between the study and test phases of a voice‐matching task (Stevenage et al., 2013). When examining performance using this interference methodology, face recognition remained strong despite the introduction of distractor faces between study and test. However, voice recognition was significantly and negatively affected by the introduction of distractor voices, suggesting, once again, that voice recognition was weaker, and more susceptible to factors that affected performance, compared with face recognition.

### 1.2 Consideration of a distinctiveness advantage

Taken together, a substantial body of work now exists to suggest that the voice is measurably weaker as a cue to identity compared with the face. This said, the examination of averaged levels of performance across a voice set may mask an important factor—the distinctiveness of one voice compared with another. In this regard, evidence is emerging to indicate a distinctiveness advantage during voice processing. When considering “familiar” voice recognition for example, Skuk and Schweinberger (2013) revealed that 12th graders were better able to recognise the voices of 20 of their classmates when those voices were distinctive rather than typical. In fact, a substantial correlation existed (*r* = 0.687) between recognition and rated distinctiveness.

In a similar vein, Foulkes and Barron (2000) asked 10 friends, and two foils to record an 8‐ to 10‐s scripted answerphone message. The 10 friends then attempted to recognise themselves and one another from the resultant 12 voice clips. Voice recognition varied substantially. However, as above, performance was significantly better when voices were more distinctive in terms of pitch and pitch variation.

Barsics and Brédart (2012b) took a slightly different approach by examining distinctiveness effects for celebrity voices. They asked participants to make a familiarity judgement to 64 celebrity or noncelebrity voices before providing episodic details of a previous encounter, plus a name or other biographic information. In keeping with the previous results, Barsics and Brédart noted better recognition of celebrity voices, and better retrieval of semantic information, when voices were distinctive than when typical.

Distinctiveness effects have also been noted when processing “unfamiliar” voices; however, here, the studies have used a broad variety of methods, and the results have not always been clear. For example, Yarmey (1991) asked participants to listen to a 36‐s monologue from a single unfamiliar speaker within the context of a fictitious kidnapping scenario. Following presentation, participants provided a description of the voice either immediately, or after a delay of a day or a week. The results suggested that the descriptions of a typical voice were substantially affected by delay. However, descriptions of a distinctive voice showed remarkable consistency even after a week, suggestive of a distinctiveness advantage with unfamiliar voices.

Mullenix et al. (2009) extended this work by using a voice recognition task, again with a single unfamiliar speaker rated as either typical or distinctive. They asked participants to engage in a word classification task to spoken words before completing a surprise voice recognition test a week later for the voice of the speaker. The recognition test took the form of an old/new matching task, with “old” clips being spoken by the target, but with “new” clips being spoken by typical and by distinctive foil speakers. Interestingly, the results did not suggest a distinctive advantage when recognising the target speaker. However, they did indicate a significantly higher error rate to “new” voices when the original target had been typical than when distinctive, and this was primarily due to confusions between the typical target and typical foils. As such, Mullenix et al. (2009) demonstrated a distinctiveness advantage with unfamiliar voices, not through better recognition of the distinctive target but through fewer false recognitions of foils.

Using a very different approach, Sauerland, Sagana, and Otgaar (2013) conducted a choice blindness task in which participants were asked to listen to three pairs of voices and to choose one from each pair according to a predefined criterion. Following each selection, the chosen voice was then represented for further consideration. However, on one critical trial, the chosen voice was switched with the nonchosen voice. A failure to spot the switch was termed “choice blindness.” Sauerland et al. (2013) noted that the incidence of choice blindness was significantly reduced when the voices in the pair were less similar to one another. In other words, participants noticed the switch more readily when the foil was very different from the chosen target, possibly because they differed significantly on rated distinctiveness.

In a more recent and novel cross‐modal study, Bülthoff and Newell (2015) asked participants to learn face–voice pairs, with half the faces paired with a distinctive voice (*n* = 12) and half with a typical voice (*n* = 12). In both a between‐participants design (Experiment 1a) and a within‐participants design (Experiment 2), the results demonstrated better subsequent recognition of the face when it had been paired with a distinctive sounding voice than with a typical sounding voice. The authors suggested that the distinctiveness of the voice made the face more distinctive and thus improved face memory. However, the results could also be interpreted in the context of multimodal person perception to which the characteristics of both the voice and the face contributed. Either way, the results suggested a unique form of distinctiveness advantage in which vocal distinctiveness facilitated subsequent person perception from the face.

In evaluating these results, it is worth noting that faces were arbitrarily paired with voices rather than being paired with their own (distinctive or typical) voices, conferring both a strength and a weakness to the design. The strength was that a common set of faces could be paired with distinctive and typical voices in a counterbalanced fashion to control item effects. However, the weakness generated by this design was that the arbitrary pairing of faces with voices could have generated mismatch effects, especially when voices were distinctive (i.e., a female voice was matched with a male face, and a Japanese speaker was matched with a Caucasian face). As such, the apparent vocal distinctiveness effect demonstrated by Bülthoff and Newell (2015) is perhaps open to interpretation.

Against this backdrop, a review of the literature suggested only one study that demonstrated a clear and direct distinctiveness advantage when recognising unfamiliar voices. This is provided by Sørensen (2012) who examined unfamiliar voice recognition by means of a delayed lineup task. Within this study, distinctiveness was operationalised based on a measure of fundamental frequency, and the results showed a distinctiveness advantage, through superior recognition performance when the voice sounded distinctive (74%) rather than typical (56%).

Taken as a whole, distinctiveness effects when processing “unfamiliar” voices have been examined using an imaginative range of methodologies. However, the results have not always clearly indicated better recognition of distinctive versus typical targets (see Mullenix et al., 2009). Additionally, generalisation of the distinctiveness advantage when processing unfamiliar voices has, at times, been limited by the use of one or, relatively, few targets (Mullenix et al., 2009; Sørensen, 2012; Yarmey, 1991; cf. Bülthoff & Newell, 2015). As such, the evidence for a distinctiveness advantage when recognising unfamiliar voices would benefit from replication and extension, and this is the purpose of Experiment 1.

Experiment 1 tests for a distinctiveness advantage with unfamiliar voices using a sequential same/different matching task. This method is favoured over an old/new recognition task due to the potential for interference effects in the latter task when presenting lists of voices at study and at test. However, the sequential nature of the task does impose a memory demand on the participants, given that the vocal information naturally unfolds over time. Nevertheless, previous studies using a sequential same/different task have shown performance levels that avoid both floor and ceiling effects (see Stevenage et al., 2013). Experiment 1 also uses a relatively large voice set to test the generalisability of previous results. Based on distinctiveness effects within the face recognition field, and the available results in the voice recognition field, it was predicted that unfamiliar voices would be recognised better when distinctive than when typical.

Experiment 1 A distinctiveness advantage in unfamiliar voice recognition

### 1.3 Design

A 2 × 2 within‐participants design was used in which vocal distinctiveness (distinctive and typical) and trial type (“same” and “different”) were varied within a sequential matching task. The participants heard two voice clips one after the other and were asked to decide whether the two clips came from the “same” speaker or from “different” speakers. Their accuracy and self‐rated confidence on “same” and “different” trials represented the dependent variables.

### 1.4 Participants

A total of 72 participants (54 females) took part in return for course credit or a small monetary payment. Their ages ranged from 19 to 35 years (*M* = 22.53, *SD* = 3.51) and all participants reported normal hearing and a lack of familiarity with the speakers.

### 1.5 Materials

A total of 117 speaker samples were collected for the purposes of this study. For all speakers, two clips were recorded so that the clips at study and at test were not identical during a “same” trial. In the study clip, the speaker said the phrase, “The smell of freshly ground coffee never fails to entice me into the shop” (mean duration = 5 s). In the test clip, they said the phrase, “The length of her skirt caused the passers‐by to stare” (mean duration = 4 s). Both phrases were created to provide phonetic richness when exploring speaker identification and were drawn from corpus of phrases used in the FRL2011 database (UK Home Office Centre for Applied Science and Technology).

The 117 speakers were designated as “distinctive” or “typical” according to the ratings of six independent judges. All ratings were made on a 7‐point scale (1 = *not at all distinctive*; 7 = *very distinctive*) and were obtained by asking the judges to imagine that they were in a noisy environment, such as a party, and to indicate how much each voice would stand out against the other voices. These instructions were modelled on those used to judge facial familiarity (“How much would this face stand out at a busy railway station?”; Valentine & Bruce, 1986). Based on these ratings, 32 distinctive voices (average distinctiveness for each ≥5) and 32 typical voices (average distinctiveness for each ≤3.5) were selected, with an equal number of male and female speakers in each set. In terms of the distinctiveness ratings, excellent agreement was indicated across the judges (Cronbach's α = 0.96), and an independent samples *t* test confirmed that the two sets of voices differed significantly in terms of their rated distinctiveness (distinctive set: *M* = 6.07, *SD* = 0.74; typical set: *M* = 2.46, *SD* = 0.65; *t*_(62)_ = 20.72, *p* < 0.001).

In addition to the target voices described above, the voices of 16 males and 16 females of intermediate distinctiveness (distinctiveness rating = 3.99, *SD* = 0.99) were selected to act as foils in the “different” trials. Given their intermediate level of distinctiveness, the foils differed on rated distinctiveness compared with both the distinctive targets (*t*_(62)_ = 9.46, *p* < 0.001) and typical targets (*t*_(32)_ = 7.27, *p* < 0.001). As a group, these foils were matched to the set of targets on sex, and on similarity of perceived pitch, according to the judgements, by ear, of the experimenters. Subsequent analysis of F0 (as determined using Praat6039 for Windows) confirmed that the foils did not differ from either the typical or the distinctive targets in terms of F0 (typical females: *t*_(30)_ = 1.22, *p* = 0.232; typical males: *t*_(30)_ = 0.48, *p* = 0.635; distinct females: *t*_(30)_ = 0.45, *p* = 0.659; distinct males: *t*_(30)_ = 0.37, *p* = 0.716). Consequently, although the individual target voices may have varied in terms of pitch, particularly in the case of distinctive targets, the population of target voices did not stand out from the population of foil voices used.

From these stimuli, 16 “same” trials and 16 “different” trials were constructed, with eight distinctive and eight typical voices contributing to each set. The “same” trials consisted of a target speaker uttering Phrase 1, and then the same speaker uttering Phrase 2. The “different” trials consisted of a target speaker uttering Phrase 1 and a same‐sex foil speaker uttering Phrase 2. Finally, the assignment of target voices to “same” and “different” trials was counterbalanced so that each voice was heard equally often in a “same” trial and a “different” trial across the participant population.

The trials were presented, and data were recorded via Superlab Pro 4.5.4 (Cedrus, released 2012) via a DELL PC laptop (with an Intel i5 core and a 64‐bit operating system) running Windows XP. All written instructions were presented via the 14″ colour screen laptop monitor, but sound was presented via outer‐ear Pro‐Luxe PRO‐40 Hi‐Fi headphones with a frequency response of 20 Hz to 20 KHz. Sound volume was adjustable via the computer settings to ensure optimal listening conditions.

### 1.6 Procedure

All participants were tested individually within a quiet testing cubicle. After providing informed consent, a practice phase was presented in which the participants were asked to press S in response to the word “same” and D in response to the word “different” as it appeared on the screen. A total of 16 trials enabled the participants to map the correct key to each response, and feedback was provided.

Following this, eight further practice trials were presented to introduce the participants to the format of the experimental trials. Instead of using voice clips, these practice trials used words. Following a “next trial” prompt, a target word was presented for 500 ms after which participants gave a rating from 1 to 7 for “pleasantness.” This ensured that the participants attended to the target. After a 5‐s gap, a second word was presented and remained on screen until the participant indicated whether it was the same (S) or different (D) to the target word seen previously. Feedback was again provided.

A self‐paced break followed during which the participants could ask for clarification of the task as required. After this, a randomised sequence of 32 experimental trials (16 “same” and 16 “different”) was presented, and no further feedback was available. All trials followed an identical format consisting of a “next trial” prompt (250 ms), a blank screen (100 ms), and the presentation of the target voice clip. The participants rated this clip for vocal attractiveness using a 7‐point scale, again as a way of ensuring attention to the target. An interstimulus interval of 16 s followed so that the matching task was not too easy. Finally, a second voice clip was presented, along with the on‐screen question, “same or different?” The participants indicated their response by pressing S for “same” and D for “different,” and the emphasis was on accuracy over speed. Finally, the participants indicated their confidence in their answer by pressing a numbered key from 1 (*not at all confident*) to 7 (*very confident indeed*).

Following completion of the task, the participants were thanked and debriefed, and the entire task lasted no more than 30 min.

## 2 EXPERIMENT 1: RESULTS AND DISCUSSION

The data from one participant were excluded through identification as an outlier reflecting poor mean performance on the easiest trials (with distinctive stimuli). The data from 71 participants remained. Given the use of a same/different task with a dichotomous response, the data were explored in line with the signal detection framework (Green & Swets, 1966). Accordingly, the accuracy scores for “same” and “different” trials were combined to yield primary measures of sensitivity of discrimination (*d′*) and response bias (*C*). The analysis of accuracy and confidence on “same” and “different” trials provided a secondary analysis.

### 2.1 Sensitivity of discrimination and response bias

Sensitivity of discrimination (*d′*) for distinctive and for typical voices is summarised in Table 1 along with a measure of response bias (*C*). Taking sensitivity of discrimination first, a paired samples *t* test was used to determine whether vocal distinctiveness had any effect on performance. This revealed a significant difference (*t*_(70)_ = 5.80, *p* < 0.001) supporting the prediction of a distinctiveness advantage. In contrast, when considering response bias (*C*), no effect of vocal distinctiveness emerged (*t*_(70)_ < 1, *p* = 0.742). In fact, one‐sample comparisons to zero revealed no bias in responding, either overall (*t*_(70)_ = 1.29, *p* = 0.20) or for distinctive and typical stimuli when taken separately (both *t*s_(70)_ < 1.17, *p* > 0.244). These results suggested that a distinctiveness advantage was demonstrable when recognising a large set of unfamiliar voices, with this being shown through sensitivity of discrimination rather than response bias.

**Table 1:** Mean sensitivity of discrimination (*d′*) and response bias (*C*), together with accuracy and self‐rated confidence on “same” and “different” trials on a same/different voice‐matching task with distinctive and typical voices in Experiment 1

	Distinctive	Typical
Sensitivity of discrimination (*d′*)	2.66 (1.24)	1.56 (1.26)
Response bias (*C*)	−0.07 (0.59)	−0.10 (0.69)
Accuracy on “same” trials	0.87 (0.12)	0.74 (0.22)
Accuracy on “different” trials	0.82 (0.19)	0.70 (0.22)
Confidence on “same” trials (/7)	5.28 (0.82)	4.57 (1.00)
Confidence on “different” trials (/7)	5.05 (1.14)	4.38 (1.19)

*Note*. Standard deviation in parentheses.

### 2.2 Accuracy of performance

Accuracy of performance is summarised in Table 1 for both “same” and “different” trials separately. This was examined by means of a 2 × 2 repeated‐measures analysis of variance (ANOVA) in which the effects of both vocal distinctiveness (distinctive and typical) and trial type (“same” and “different”) were explored. Importantly, there was a main effect of distinctiveness, *F*(1, 70) = 40.09; *p* < 0.001; η^2^_G_ = 0.09; *MSE* = 0.03, with performance being better for distinctive than for typical voices. The analysis revealed no main effect of trial type, *F*(1, 70) = 2.95; *p* = 0.09; η^2^_G_ = 0.01; *MSE =* 0.05. Moreover, there was no interaction between distinctiveness and trial type, *F*(1, 70) < 1; *p* = 0.80; η^2^_G_ < 0.01; *MSE* = 0.02), indicating that the distinctiveness advantage emerged for “same” and “different” trials alike.

### 2.3 Self‐rated confidence

Finally, analysis was conducted on self‐rated confidence when recognising typical and distinctive voices. These data were calculated across all trials and are summarised in Table 1. They were analysed by means of a 2 × 2 repeated‐measures ANOVA in which vocal distinctiveness (distinctive and typical) and trial type (“same” and “different”) were explored. As above, this revealed a main effect of distinctiveness, *F*(1, 70) = 69.77; *p* < 0.001; η^2^_G_ = 0.23; *MSE* = 0.49, with confidence being greater when recognising distinctive voices than when recognising typical ones. There was, however, no main effect of trial type, *F*(1, 70) = 3.88; *p* = 0.053; η^2^_G_ = 0.03; *MSE* = 0.80. Again, there was no interaction between distinctiveness and trial type, *F*(1, 70) < 1; *p* = 0.81; η^2^_G_ < 0.01; *MSE =* 0.31, indicating that confidence was greater for distinctive than typical voices in “same” and “different” trials alike.

Taken together, the data from Experiment 1 were clear in supporting the prediction of a distinctiveness advantage when recognising unfamiliar voices. This advantage was revealed in sensitivity of discrimination, accuracy for “same” and “different” trials, and in self‐rated confidence. One strength of the current study lies with the use of a large number of distinctive and typical voices, avoiding concerns that previous mixed results may have been driven by particular items within small stimulus sets. As such, this evidence sits well alongside the considerable body of work indicating a distinctiveness advantage when recognising faces (Bartlett, Hurry, & Thorley, 1984; Goldstein & Chance, 1981; Light, Kayra‐Stuart, & Hollander, 1979; Shepherd, Gibling, & Ellis, 1991; Valentine & Bruce, 1986; Winograd, 1981), as well as the findings indicating a distinctiveness advantage when recognising personally familiar or celebrity voices.

### 2.4 Accounting for distinctiveness effects using a similarity space framework

In the context of face processing, the distinctiveness advantage has been elegantly accounted for by appealing to the fact that distinctive items stand out on one or more dimensions of a face similarity space. Consequently, they suffer less confusability with near‐neighbours during a recognition task compared with their typical counterparts (Valentine, 1991). Recent work has extended the concept of a similarity space to the perception of voices, with dimensions of the space reflecting the vocal characteristics that listeners use to differentiate voices (Baumann & Belin, 2010). By extension, distinctive voices again stand out on one or more dimensions that define the voice space, leading to less confusability with vocal near‐neighbours compared with their typical voice counterparts. As such, Experiment 1 provides a valuable addition to the empirical evidence for a distinctiveness advantage when recognising unfamiliar voices, suggesting a robust and replicable effect that can be readily accounted for within a similarity‐based voice space framework.

Experiment 2 A distinctiveness advantage under challenging conditions

The next natural question is whether vocal distinctiveness would assist the listener even when listening conditions are challenging. Such a prediction may follow by extension of the voice space framework. In this context, if listening conditions are challenging, the error when encoding a voice may be expected to increase, reducing the likelihood of a match between a voice and its previously stored representation. This additional error may be more likely to affect typical voices than distinctive voices, given that typical voices are more confusable at the outset. As a result, it may be predicted that distinctive voices would retain a processing advantage even when presented under challenging listening conditions.

Two studies are of relevance to this question in as much as they suggest quite contradictory findings. The first study is provided by van Lancker, Kreiman, and Emmorey (1985), who tested familiar voice recognition under three discrete conditions. First, participants listened to 2‐s voice clips belonging to 45 celebrities before indicating whether each voice was familiar (or not) from an unlimited set (Task 1). Following this, participants listened to a new set of 2‐s voice clips for the same celebrities, before indicating the speakers' identity by selecting one of six possible names (Task 2). Participants were able to recognise nearly 27% of targets when presented in an unlimited set, and nearly 70% of targets when presented in a six‐alternate forced‐choice task. Of most interest, however, was the performance in a final condition in which participants listened to 4‐s clips played backwards, before again indicating speaker identity from six names (Task 3). Remarkably, participants remained able to recognise over 57% of targets in the 6AFC task despite their temporal reversal. Notably, performance on these backwards voices varied substantially across the targets, with some targets being “equally recognisable” when played backwards as when played forwards. The authors considered that these unanticipated item effects may have been driven by variation in the distinctiveness of the target voices, suggesting that distinctiveness may provide an advantage when processing voices under difficult or unusual listening conditions.

In direct contrast are the findings of Orchard and Yarmey (1995). As in Yarmey's (1991) earlier work, Orchard and Yarmey asked participants to listen to either a distinctive or a typical target voice presented in the context of a fictitious kidnapping scenario. Two days later, participants were asked to identify the target from a six‐person target‐present or target‐absent lineup. Several factors were varied including whether the target spoke normally or in a whisper and whether the voice at lineup was of the same format (normal or whisper) to the voice at study. The results suggested that performance was significantly impaired by whispering and by a change in speech style, in both target‐present and target‐absent lineups. Of more interest, however, performance was significantly affected by the distinctiveness of the target voice, but surprisingly, this indicated a trend for typical sounding voices to be better recognised—a distinctiveness disadvantage. This appeared to be mediated by several variables including whether the speaker was whispering and whether the listener felt confident in their recognition. As such, the evidence regarding a distinctiveness advantage under difficult listening conditions remains unclear. Experiment 2 was designed to address this issue.

Within Experiment 2, challenging listening conditions were introduced through either changing the word order within a sentence to create a nonsense clip or through temporally reversing the voice clip. Similar manipulations have been used with faces as ways to disrupt facial processing. In such studies, the scrambling of features within an otherwise upright face, or the inversion of the face entirely, has been thought to disrupt the ability to process the critical relationships between features (see Tanaka & Farah, 1993). Although it cannot be assumed that nonsense speech or temporal reversal have the same disruptive effect on voices as scrambling and inversion have on faces, these manipulations have been used to good effect when making voice processing difficult (see Goggin, Thompson, Strube, & Simental, 1991, Expt 4; van Lancker et al., 1985).

Manipulation here through the creation of a nonsense clip, or through temporal reversal, has the advantage of introducing a cognitive challenge to the listening task whilst leaving the paralinguistic properties of the stimuli unaffected. To the extent that vocal distinctiveness may be carried in these vocal properties rather than in features associated with the utterance, the distinctiveness of the voice was unchanged by the manipulation of task difficulty. Given this, if vocal distinctiveness is effective in protecting voice recognition abilities as predicted, then the recognition of distinctive voices should be superior to that of typical voices, even under these challenging listening conditions.

### 2.5 Design

A 3 × 2 mixed design was used in which listening condition (forwards, nonsense, and backwards) was manipulated between participants, and vocal distinctiveness (distinctive and typical) was manipulated within participants. As in Experiment 1, voice recognition was examined through a sequential same/different matching task, and accuracy and self‐rated confidence represented the dependant variables.

### 2.6 Participants

A total of 48 participants (37 females) took part in return for course credit. The participants were randomly assigned to one of three listening conditions such that they heard speech at study that was either played forwards (*n* = 16, 12 females); in a nonsense order (*n* = 16, 12 females); or backwards (*n* = 16, 13 females). The participants' ages ranged from 15 to 60 years (*M* = 23.9 years, *SD* = 10.2), and all participants had normal, or corrected‐to‐normal, hearing. The participants reported no familiarity with the stimuli, and none had taken part in the previous experiment.

### 2.7 Materials

Bespoke stimuli were used for this experiment, consisting of 60 speakers drawn from the same demographic population as the participants in terms of age range and accent. All speakers were recorded uttering a study phrase under various conditions, along with a test phrase. Mirroring Experiment 1, the study phrase was, “The smell of freshly ground coffee never fails to entice me into the shop.” To provide a nonsense version of this study phrase, the adjectives and nouns were repositioned within the sentence (“The shop of ground fails smell never coffee into the me freshly to entice”). The speakers practiced this nonsense phrase prior to recording until they were able to utter it with a cadence and phrasing that felt natural. To provide a temporally reversed (backwards) version of the study phrase, the “reverse” function within Audacity 2.0.5 was used, resulting in a clip that was incomprehensible whilst still preserving the acoustic properties of the speaker. Finally, and again mirroring Experiment 1, the speakers were recorded uttering a separate test phrase (“The length of her skirt caused the passers‐by to stare”). This ensured that the study and test phrases were not identical during a “same” trial.

Using the standard study phrase played forwards, all speakers were rated for their vocal distinctiveness by the experimenters using a 7‐point scale, where 1 = *not at all distinctive* and 7 = *very distinctive indeed*. On the basis of these ratings, 16 distinctive voices (*M* = 5.69, *SD* = 0.68) and 16 typical voices (*M* = 3.50, *SD* = 0.48) were selected as targets. In terms of the distinctiveness ratings, agreement between the raters was again good (Cronbach's α = 0.82), and the two voice sets differed significantly on vocal distinctiveness (*t*_(30)_ = 10.49, *p* < 0.001).

The 32 target voices were then paired to construct 16 “same” trials (eight distinctive and eight typical) and 16 “different” trials (eight distinctive and eight typical). The “same” trials were constructed by pairing a study clip from one speaker with a test clip of the same speaker, whereas the “different” trials were constructed by pairing a study clip from one speaker with a test clip from a same‐sex foil speaker drawn from the remaining voices. Similarity ratings by the experimenters on a 7‐point scale (1 = *not at all similar* and 7 = *very similar indeed*) confirmed that the similarity of distinctive and typical targets to their respective foils was matched (distinctive similarity: *M* = 5.87, *SD* = 0.64; typical similarity: *M* = 5.87, *SD* = 0.79; *t*_(14)_ < 1, *ns*). This ensured that the “different” trials did not represent a trivially easy task for one or other voice set.

The voices trials were presented, and data were recorded using SuperLab Pro 4.5.4 via a DELL PC laptop (with an Intel i5 core and a 64‐bit operating system) running Windows 7. As in Experiment 1, all written instructions were presented via the 14″ colour screen laptop monitor, but sound was presented via outer‐ear Pro‐Luxe PRO‐40 Hi‐Fi headphones with a frequency response of 20 Hz to 20 KHz. Sound volume was adjustable via the computer settings to ensure optimal listening conditions.

### 2.8 Procedure

The participants were tested individually within a quiet experimental cubicle. Following explanation of the task, and the indication of informed consent, the participants completed a set of practice trials during which they were required to press “S” or “D” to the words “same” or “different” as they appeared on the screen. This stage enabled the participants to map the correct keyboard key to each response.

After a self‐paced break, the 32 experimental trials (16 “same” and 16 “different”) were presented in a random order. All trials took the same format beginning with a “next trial” prompt (250 ms) to encourage the participants to orient towards the task. This was followed by the presentation of the study voice clip for 4 s. The voice was either distinctive or typical and was heard in either the forwards, nonsense, or backwards format depending on the condition to which the participant had been assigned. In contrast to Experiment 1 in which a 16‐s gap was used, the present study adopted only a 4‐s gap between study and test clips. This change reflected a desire to avoid floor effects associated with poor performance in the most challenging of the listening conditions. Following this 4‐s gap, the test clip was played, and the participants' task was to indicate whether it was the “same” speaker or a “different” speaker to the one heard at study. The participants responded by pressing “S” or “D,” respectively. Finally, they indicated their confidence in their answer by pressing a numbered key from 1 (*not at all confident*) to 7 (*very confident indeed*).

The entire experiment lasted approximately 25 min, after which the participants were thanked and debriefed.

## 3 EXPERIMENT 2: RESULTS AND DISCUSSION

As in Experiment 1, the accuracy data for “same” and “different” trials were combined to provide measures of sensitivity of discrimination (*d′*) and bias (*C*). Primary analyses are reported on these measures, with secondary analyses provided using accuracy and confidence on “same” and “different” trials.

### 3.1 Sensitivity of discrimination (*d*′)

Sensitivity of discrimination (*d*′) and response bias (*C*) when recognising distinctive and typical sounding voices are summarised in Table 2 when voices were played in forwards, nonsense, and backwards formats at study. A 2 × 3 mixed ANOVA on sensitivity of discrimination revealed a significant main effect of distinctiveness, *F*(1, 45) = 15.71; *p* < 0.001; η^2^_G_ = 0.11; *MSE* = 0.64, with performance being better for distinctive than for typical voices, as predicted. In addition, there was a significant main effect of listening condition, *F*(2, 45) = 43.36; *p* < 0.001; η^2^_G_ = 0.55; *MSE* = 1.11, with repeated contrasts indicating equivalence between forwards and nonsense listening conditions (*p* = 0.80) but showing a substantial reduction in performance between nonsense and backwards conditions (*p* < 0.001). The interaction between distinctiveness and listening condition was not significant, *F*(2, 45) < 1; *p* = 0.57; η^2^_G_ < 0.01; *MSE* = 0.64, suggesting that distinctiveness provided an overall advantage in each listening condition.

**Table 2:** Mean sensitivity of discrimination (*d′*) and response bias (*C*), together with accuracy and self‐rated confidence (with standard deviation) when recognising distinctive and typical voices under forwards, nonsense, and backwards listening (Experiment 2)

	Distinctive	Typical
Forwards		
Sensitivity of discrimination (*d*′)	3.22 (1.23)	2.70 (1.08)
Response bias (*C*)	−0.04 (0.36)	−0.19 (0.61)
Accuracy on “same” trials	0.91 (0.10)	0.88 (0.17)
Accuracy on “different” trials	0.91 (0.10)	0.83 (0.13)
Confidence on “same” trials (/7)	6.10 (0.53)	5.80 (0.63)
Confidence on “different” trials (/7)	6.20 (0.81)	5.49 (0.45)
Nonsense		
Sensitivity of discrimination (*d′*)	3.29 (1.08)	2.77 (0.58)
Response bias (*C*)	0.18 (0.47)	−0.39 (0.63)
Accuracy on “same” trials	0.88 (0.13)	0.92 (0.12)
Accuracy on “different” trials	0.94 (0.09)	0.80 (0.13)
Confidence on “same” trials (/7)	6.01 (0.78)	5.72 (0.67)
Confidence on “different” trials (/7)	6.07 (0.78)	4.92 (0.83)
Backwards		
Sensitivity of discrimination (*d′*)	1.32 (0.73)	0.43 (0.70)
Response bias (*C*)	−0.08 (0.36)	−0.11 (0.46)
Accuracy on “same” trials	0.76 (0.12)	0.61 (0.22)
Accuracy on “different” trials	0.70 (0.21)	0.54 (0.19)
Confidence on “same” trials (/7)	3.50 (1.50)	2.87 (1.47)
Confidence on “different” trials (/7)	3.70 (1.61)	3.05 (1.53)

### 3.2 Bias (*C*)

A 2 × 3 mixed ANOVA was also conducted on response bias (*C*). This revealed a main effect of distinctiveness, *F*(1, 45) = 7.35; *p* = 0.009; η^2^_G_ = 0.06; *MSE* = 0.20, in which distinctive voices attracted significantly less bias than typical ones. There was no significant effect of listening condition overall, *F*(2, 45) < 1; *p* = 0.992; η^2^_G_ < 0.01; *MSE* = 0.29. However, a significant interaction between distinctiveness and listening condition did emerge, *F*(2, 45) = 3.32; *p* = .045; η^2^_G_ = 0.06; *MSE* = 0.20. Tests of simple main effects revealed a significant reduction in response bias for distinctive over typical voices for nonsense speech, *F*(1, 45) = 13.08; *p* = 0.001; η^2^_G_ = 0.15; *MSE* = 0.22, but not when speech was played forwards, *F*(1, 45) < 1; *p* = 0.392; η^2^_G_ = 0.01; *MSE* = 0.22, or backwards, *F*(1, 45) < 1; *p* = 0.879; η^2^_G_ < 0.01; *MSE* = 0.15, when bias was minimal.

### 3.3 Accuracy

Accuracy of performance when recognising distinctive and typical voices is summarised in Table 2 in each of the experimental conditions. This was examined using a 3 × 2 × 2 mixed ANOVA in which listening condition (forwards, nonsense, and backwards); distinctiveness (distinctive and typical); and trial type (“same” and “different”) were varied. The analysis indicated no main effect of trial type, *F*(1, 45) = 2.83; *p* = 0.099; η^2^_G_ = 0.02; *MSE* = 0.03, and no interaction of trial type with any other variable (all *F*s < 3.53, *p* > 0.067, η^2^_G_ < 0.02, *MSE* = 0.02). Thus, performance was equivalent across “same” and “different” trials within this experiment. More importantly, the results indicated a significant main effect of distinctiveness, *F*(1, 45) = 30.50; *p* < 0.001; η^2^_G_ = 0.08; *MSE* = 0.01, and a significant main effect of listening condition, *F*(2, 45) = 40.51; *p* < 0.001; η^2^_G_ = 0.37; *MSE* = 0.03. As in the analysis of sensitivity of discrimination above, these confirmed that voice recognition was significantly better for distinctive than for typical voices but was also significantly impaired, as the message became more difficult to process.

Somewhat surprisingly, and in contrast to the analysis of sensitivity of discrimination, a significant interaction emerged between distinctiveness and listening condition, *F*(2, 45) = 4.60; *p* = 0.015; η^2^_G_ = 0.03, *MSE* = 0.01. In line with the a priori expectations, tests of simple main effects confirmed this to be due to a significant distinctiveness advantage in all conditions, forwards: *F*(1, 45) = 4.73; *p*_1‐tailed_ = 0.018; η^2^_G_ = 0.06, *MSE* = 0.01; nonsense: *F*(1, 45) = 3.03; *p*_1‐tailed_ = 0.045, η^2^_G_ = 0.04; *MSE* < 0.01; backwards: *F*(1, 45) = 31.95; *p*_1‐tailed_ < 0.001; η^2^_G_ = 0.23; *MSE* = 0.01, but the effect was somewhat smaller in the nonsense condition.

### 3.4 Confidence

Self‐rated confidence is summarised in Table 2 and was analysed as above using a 3 × 2 × 2 mixed ANOVA. Analysis across all trials revealed a broadly similar pattern of performance to that above. There was no main effect of trial type, *F*(1, 45) = 1.77; *p* = 0.19; η^2^_G_ < 0.01; *MSE* = 0.24. However, there was a significant main effect of distinctiveness, *F*(1, 45) = 84.13; *p* < 0.001; η^2^_G_ = 0.09; *MSE* = 0.22, and a significant main effect of listening condition, *F*(2, 45) = 38.82; *p* < 0.001; η^2^_G_ = 0.59; *MSE =* 3.49. Two of the two‐way interactions were significant, trial type x listening condition: *F*(2, 45) = 5.20; *p* = 0.009; η^2^_G_ = 0.01; *MSE =* 0.24; trial type x distinctiveness: *F*(1, 45) = 11.38; *p* = 0.002; η^2^_G_ = 0.01; *MSE* = 0.19, but the remaining two‐way interaction between distinctiveness and listening condition was not significant, *F*(2, 45) < 1, *p* = 0.446, η^2^_G_ < 0.01, *MSE* = 0.22. Finally, a small but significant three‐way interaction emerged between all variables, *F*(2, 45) = 3.65; *p* = 0.034; η^2^_G_ < 0.01; *MSE* = 0.19).

Further examination of this three‐way interaction through analysis of the simple main effects revealed a main effect of distinctiveness within each listening condition, forwards: *F*(1, 45) = 18.66; *p* < 0.001; η^2^_G_ = 0.02, *MSE* = 0.22; nonsense: *F*(1, 45) = 37.35; *p* < 0.001; η^2^_G_ = 0.21; *MSE* = 0.24; backwards: *F*(1, 45) = 29.75; *p* < 0.001; η^2^_G_ = 0.05; *MSE* = 0.20, which interacted with trial type only in one condition, nonsense: *F*(1, 45) = 15.22; *p* < 0.001; η^2^_G_ = 0.09; *MSE* = 0.21. “Pairwise” comparisons in the nonsense condition tested the a priori prediction of a distinctiveness advantage. These nevertheless revealed significantly greater confidence for distinctive than typical voices in both same trials, *t*(15) = 2.21, *p* = 0.043, and different trials, *t*(15) = 5.80, *p* < 0.001, suggesting that the three‐way interaction may reflect noise in the data.

Taken together, these results were interesting in several regards. First, they provided support for the prediction that performance on a difficult voice recognition task would be facilitated by the distinctiveness of the voice. This was demonstrated in terms of sensitivity of discrimination, accuracy, and in terms of metacognitive judgements of confidence in decision‐making. This was most apparent in the temporally reversed condition when voice recognition became significantly impaired. As such, the present results complemented those of van Lancker et al. (1985), who suggested that distinctiveness may support voice recognition even under temporal reversal. However, the present results go one step further by providing an a priori test rather than a post hoc explanation, of the importance of distinctiveness under difficult listening conditions.

This said, a subtlety emerged in the manipulation of task difficulty that had not been anticipated. Indeed, reordering the words to create nonsense speech had relatively little effect on accuracy of voice recognition performance overall. Moreover, the distinctiveness advantage in the “nonsense” condition was significant but was relatively weak, and some evidence emerged of a shift in response bias in this condition. This was surprising, as it has been assumed that voice recognition from nonsense speech would represent a more challenging task compared with the baseline condition.

In accounting for the results in the “nonsense” condition, it is possible that the absence of a clear impact on performance in this condition reflected the relatively low power within the current design as a whole. Certainly, the current results would benefit from replication using a greater number of participants. However, it may also be possible to explain the results in the “nonsense” condition with reference to potential strategies that the participants could have adopted. In particular, it is possible that they concentrated on the common start of the clip (“The shop of ground …”) and disregarded the remainder of the nonsense phrase. This may have enabled the participants to perform well despite the increasing bizarreness in the nonsense phrase, as it unfolded. It should also be noted that the nonsense phrase was represented with every speaker at study, and participants reported habituation to its bizarreness as the study wore on. As a consequence, accuracy remained relatively high in this condition (however, see Appendix A for analysis of this point).

Additionally, it is possible that participants in the “nonsense” condition were able to perform well because they disregarded the bizarre words entirely and instead utilised the melody contour (ups and downs) in the nonsense clip. Indeed, the fact that the speakers within this study had practiced the nonsense phrase meant that they could utter it with a near‐natural cadence and intonation and these prosodic characteristics may have minimised the impact of the nonsense manipulation. By contrast, it is notable that when Goggin et al. (1991) generated nonsense clips by digitally cutting and reordering the voice clips, their clips did not retain a natural prosody, and a reduction in voice recognition performance was noted (see Goggin et al., 1991).

In considering the importance of the melody contour within speech, it is conceivable that a rich melody contour may be considered an aspect of vocal distinctiveness. Some interesting work on processing the melody contour reveals that this is discernible by infants, nonmusicians and musicians alike, suggesting that it may be extracted automatically (see Lee, Janata, Frost, Hanke, & Granger, 2011). This said, there is some evidence to suggest that the processing of melody contours in music and in speech may differ, with the latter being far more coarse‐grained than the former (see Zatorre & Baum, 2012). As such, a musical contour explanation provides a potentially valuable interpretation of the surprisingly good performance in the “nonsense” condition but would benefit from further exploration. Moreover, given the surprising results in the “nonsense” condition, there may be value in exploring performance under a different type of challenge, whilst still holding paralinguistic properties of the voice constant. Such conditions may be provided when listening to speech amidst noise (i.e., Sumby & Pollack, 1954) or when listening to speech at low volume. Further work on this issue may be of value in addressing the weaknesses of the current “nonsense” condition.

## 4 GENERAL DISCUSSION

The results presented here have provided an effective demonstration of a distinctiveness advantage when recognising unfamiliar voices. In Experiment 1, distinctive voices were recognised with greater sensitivity of discrimination, accuracy, and confidence than their typical sounding counterparts, and as such, the prediction of a distinctiveness advantage when recognising unfamiliar voices was supported. Moreover in Experiment 2, the distinctiveness advantage remained evident despite perceptually challenging listening conditions. In comparing performance across studies, it is notable that performance in the baseline (forwards) condition of Experiment 2 appeared better, and participants appeared more confident, than in Experiment 1. This most likely resulted from the reduction in delay between study and test in Experiment 2 (from 16 to 5 s). As noted earlier, this change was important in reducing the likelihood of poor performance and thus floor effects in the disrupted listening conditions of Experiment 2. Nevertheless, it is appropriate to note this difference and to refrain from drawing a direct comparison of absolute performance levels across the studies.

Taken together, these results confirmed the findings of a diverse set of previous studies (Bülthoff & Newell, 2015; Mullenix et al., 2009; Sauerland et al., 2013; Sørensen, 2012, van Lancker et al., 1985; Yarmey, 1991). The benefit of the present results, however, was that the distinctiveness advantage was demonstrated here across a considerably larger voice set than has been utilised previously and was demonstrated across a more standard voice‐matching task under both optimal and suboptimal listening conditions.

This demonstration of a vocal distinctiveness advantage sits well with the face literature (Bartlett et al., 1984; Goldstein & Chance, 1981; Light et al., 1979; Shepherd et al., 1991; Valentine & Bruce, 1986; Winograd, 1981) suggesting value in the application of methodologies and findings across the two domains. Similarly, the demonstration of a distinctiveness advantage can be readily accommodated within a similarity space explanation. This suggests that stimuli, be they faces (Valentine, 1991) or voices (Baumann & Belin, 2010), can be arranged in a similarity space on the basis of their properties along each of the dimensions that describe the space. Typical stimuli will naturally fall towards the centre of the space and will be located in a relatively densely populated area with many near neighbours. By comparison, distinctive stimuli will, by definition, stand out on one or more of the dimension(s) that define the space and thus will fall towards the edge of the space where there are fewer near neighbours. The distinctiveness advantage has been accounted for as a natural consequence of the fact that distinctive stimuli have fewer near neighbours with which to be confused and thus can be more easily matched to a (temporary) stored representation.

The present results may hold value when considering voice processing in an applied context, and it is useful to reflect briefly on this possibility. For instance, given the current results, it is perhaps tempting to conclude that police investigators may justifiably have greater confidence in earwitness recognition when the target voice sounds distinctive rather than typical. Taken in a wider context, however, the present findings of better recognition memory for distinctive over typical voices should be tensioned with an indication of a greater risk of a “false feeling of familiarity” when voices are distinctive (see Krix, Sauerland, & Schreuder, 2017). Additionally, the performance of participants within a laboratory context may overestimate performance in more real‐world settings for a host of reasons. As such, demonstration of a distinctiveness advantage under a range of ecologically valid conditions will require further empirical testing.

Perhaps of greater importance, however, the present paper invites a careful consideration of the concept of distinctiveness, as it applies to voices. Indeed, this may represent a fruitful avenue for future work. If one adopts the statistical approach to define distinctiveness (such as that defined within models of similarity space; Valentine, 1991), then a distinctive voice is any voice that stands out for any reason relative to the set of voices under consideration. This is the approach that has been used within this paper. 1We differentiate here between distinctive of the voice (on the basis of vocal characteristics) and distinctiveness of the presentation of the voice (by scrambling for instance). In the current study, distinctiveness refers to “vocal” distinctiveness rather than unusualness created through nonstandard presentation of an otherwise typical sounding voice. Within this approach, it stands to reason that distinctiveness, by definition, is a “*relative*” rather than an “*absolute*” characteristic. Put another way, a voice that is distinctive due to an unusually low pitch (relative to some comparison set) will no longer be distinctive if all the comparison voices also have a low pitch.

Respecting this line of thought, vocal distinctiveness rests on a notable difference between a target voice and a set of comparison voices, but the type of difference is unspecified. This is the case when distinctiveness rests on a global and unspecified rating indicating that a voice “stands out within a noisy environment,” or when judging “unusualness” or difficulty to recognise a voice in a group (Krix et al., 2017). The strength of such an approach is that distinctiveness effects can be examined without constraining the basis for the distinctiveness ratings to what we may know or presume given our current understanding. The weakness of such an approach is that the basis of distinctiveness for each voice is ignored.

If, instead, one seeks to understand the particular characteristics that make a voice distinctive, it may be useful to consider those characteristics that we commonly use to distinguish one voice from another. Baumann and Belin (2010) identified pitch and formant characteristics when mapping their vocal similarity space. From this, it may be suggested that listeners judge a voice to be distinctive if it stands out on one or more of these dimensions. This definition sits well with the work of Foulkes and Barron (2000) and Sørensen (2012) who both explored voice processing when targets were distinctive in terms of pitch, or pitch variation. This said, Baumann and Belin's (2010) use of vowel sounds (“a,” “i,” and “u”) as a basis for determining their voice space may have ignored other more prosodic vocal features, which emerge as speech unfolds over time. Accordingly, distinctiveness has at times been operationalised through other characteristics such as accent or language (Bülthoff & Newell, 2015). Still, other studies have suggested that voices may be described using rich descriptors including nasality, speed, intonation, volume, tremor, and pauses (see Yarmey, 1991 for a set of descriptive ratings used). These provide an expanded set of characteristics that could serve as the basis for distinctiveness.

Adopting this line of thought would enable researchers to potentially generate distinctive versions of voices by using voices that vary naturally on some specified dimension (such as pitch or speed) or by using synthetic voices that have been manipulated to vary along a specified dimension. The effectiveness of such a manipulation will necessarily depend upon a host of factors including the extent of manipulation, the just‐noticeable differences, and the initial vocal characteristics; for a voice that is already relatively high in pitch, a further manipulation of pitch may have little impact.

One potentially promising way forward is to make use of recent developments in voice morphing software. This is capable of generating caricatures, and anti‐caricatures of voices compared with some norm or reference point (Kawahara & Matsui, 2003; Schweinberger, Kawahara, Simpson, Skuk, & Zäske, 2013). As such, a caricature may be considered to represent a distinctive version of a given voice, and an anti‐caricature may represent a typical version of the given voice, relative to a norm. In this way, distinctiveness could be varied “within” the voice, allowing for highly controlled tests of distinctiveness effects (see Rhodes, Brennan, & Carey, 1987, for a similar approach in the area of face processing). Such an approach would not enable the identification of the individual characteristics that make a voice distinctive, but it would enable the controlled manipulation of distinctiveness by exaggerating the characteristics that make “each” individual voice stands out. Future work along these lines would be valuable in providing a sophisticated test of distinctiveness effects in voices.

### 4.1 Summary

In summary, the present paper has reported on the results of two experiments, which explored a distinctiveness advantage when recognising unfamiliar voices. The results of Experiment 1 confirmed that distinctive voices were processed with greater sensitivity of discrimination, accuracy, and confidence compared with typical voices. The results of Experiment 2 extended these findings by confirming a distinctiveness advantage even under difficult listening conditions involving nonsense phrases and backwards speech. These results sit well alongside the considerable body of research suggesting a facial distinctiveness advantage. Moreover, they can be readily explained by drawing on the concept of a similarity space. Nevertheless, the use of generalised ratings of distinctiveness here did not address the issue of what makes a voice distinctive, making this an exciting avenue for future research.

## APPENDIX A

### A.1

In examining the possibility that participants habituated to the nonsense phrase across the course of the experiment, an analysis based on the responses during the first half of the experiment only suggested that performance remained high when listening to nonsense messages. In fact, there was no significant difference in *d*′ for distinctive voices, *t*(15) < 1, *p* = 0.675, or for typical voices, *t*(15) < 1, *p* = 0.752, when comparing performance across the two halves of the experiment.

Furthermore, if the data from the first half of the experiment were considered for those in the “nonsense” group alongside all the data from those in the “forwards” group and the “backwards” group, the ANOVA replicated all reported findings. There was a main effect of distinctivenes, *F*(1, 45) = 11.45; *p* = 0.001; η^2^_G_ = 0.11; *MSE* = 44.67, indicating better performance with distinctive than typical voices overall. There was again a main effect of listening condition, *F*(2, 45) = 54.07; *p* < 0.001; η^2^_G_ = 0.56; *MSE* = 48.94. Again, the interaction between distinctiveness and listening condition was not significant, *F*(2, 45) < 1; *p* = 0.761; η^2^_G_ < 0.01; *MSE* = 44.67.

Importantly for the current discussion, repeated post hoc contrasts were used to examine the main effect of listening condition. As in the full analysis, these again revealed equivalent performance when comparing the “forwards” condition to the “nonsense” condition (*p* = 0.087), but a significant reduction in performance between the “nonsense” condition and the “backwards” condition (*p* < 0.001). Consequently, these results suggest that a full account of the maintenance of performance from “forwards” to “nonsense” conditions may be more complex than a simple habituation effect.
